# Supplementary material for: A GASA Protein Family Gene, CmGEG, Inhibits Petal Growth in Chrysanthemum
Source: Int J Mol Sci. 2024 Mar 16;25(6):3367. doi: 10.3390/ijms25063367 (PMC10970651; doi:10.3390/ijms25063367)
Supplement: Supplementary file 1 [file ijms-25-03367-s001.zip › Supplementary Figures.pdf]

Supplementary Material

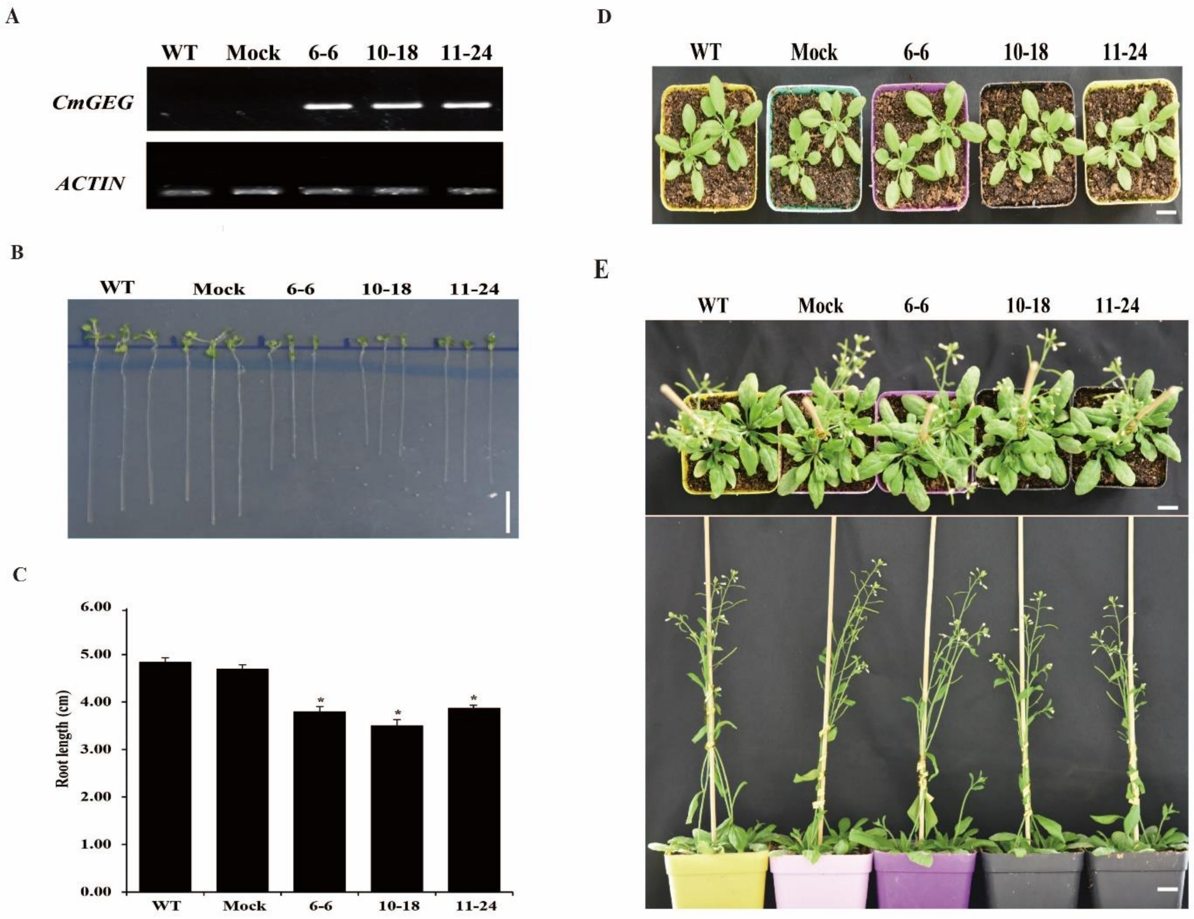

Supplementary Figure S1. Phenotypes of *CmGEG-OE* lines in *Arabidopsis*. (A) RT-PCR analysis of T_3 *CmGEG*-overexpressing transgenic lines and *AtEF1α* is the reference gene. (B) Root length phenotypes of *CmGEG-OE* lines. (C) Statistics of root length in *CmGEG-OE* lines. (D, E) Phenotype of *CmGEG-OE* lines after seedling establishment. Scale bar = 1 cm. 6-6, 10-18 and 11-24 indicate three independent homozygous lines. * $p < 0.05$.

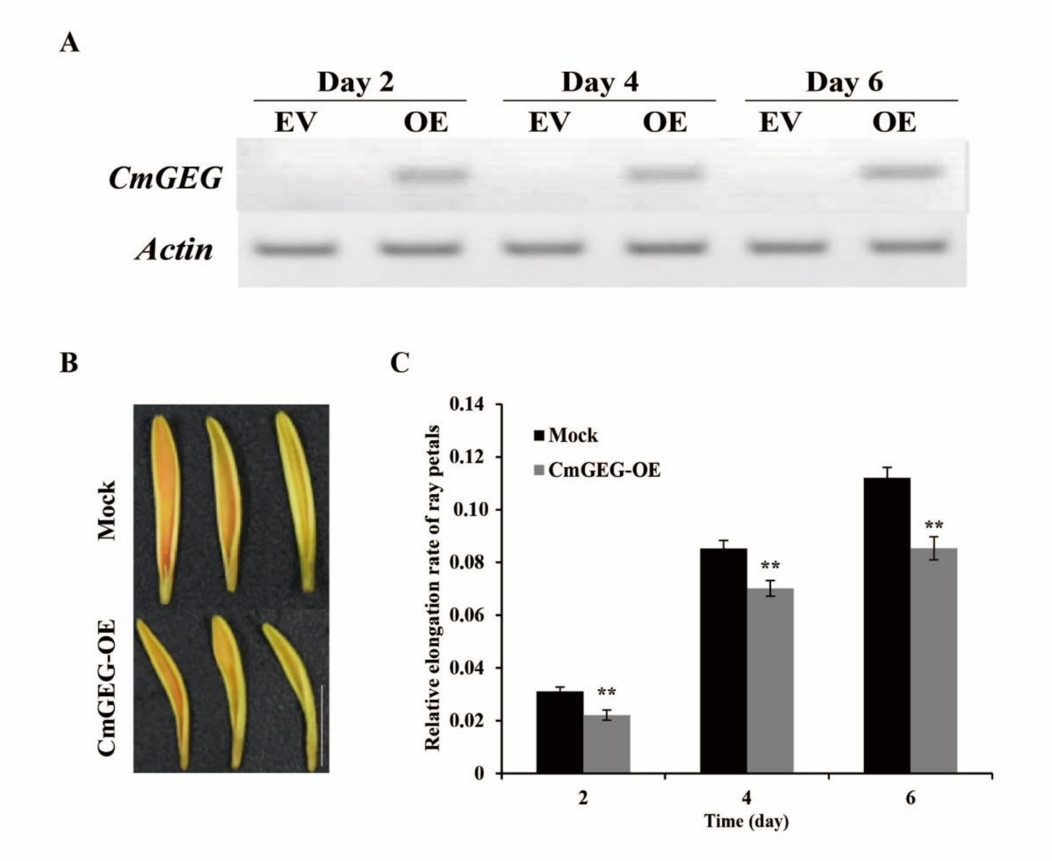

Supplementary Figure S2. *CmGEG* inhibits the elongation of ray petals in gerbera. (A) RT-PCR analysis of *CmGEG* expression levels at days 2, 4 and 6 of culture. EV: Empty Vector; OE: Overexpression. (B) Petal phenotypes of CmGEG-OE and Mock after 6 days of culturing. (C) Relative elongation rates of ray petals in CmGEG-OE and Mock. Values are the mean ± SD from three biological replicates. Scale bar = 1 cm . ** $p < 0.01$

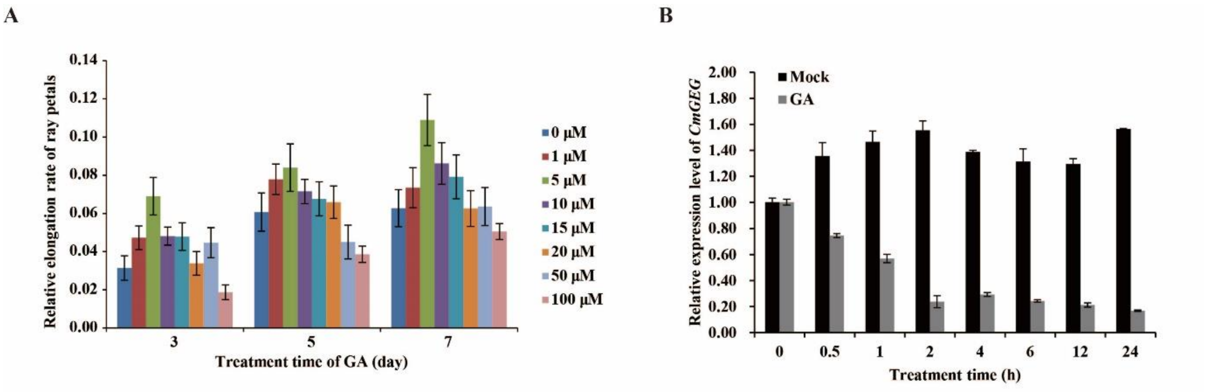

Supplementary Figure S3. Effects of GA on petal growth in chrysanthemum. (A) The relative elongation rate of petals treated with different concentrations of GA and deionized water (control) for 7 days. (B) The relative expression level of *CmGEG* in petals were detected at different time points (0, 0.5, 1, 2, 4, 8, 12, and 24 h) under the deionized water (control), GA (5 μM) treatments. Values are the mean ± SD from three biological replicates.
