# Supplementary material for: A GASA Protein Family Gene, CmGEG, Inhibits Petal Growth in Chrysanthemum
Source: Int J Mol Sci. 2024 Mar 16;25(6):3367. doi: 10.3390/ijms25063367 (PMC10970651; doi:10.3390/ijms25063367)
Supplement: Supplementary file 1 [file ijms-25-03367-s001.zip › Supplementary Table S1.pdf]

Supplementary Material

Supplementary Table S1. Primers used

Primer name	Primer sequence (5'→3')
EF1α-F	TTTTGGTATCTGGTCCTGGAG
EF1α-R	CCATTCAAGCGACAGACTCA
CmGEG-F1	ATGGCCATCTCGAAGCCTTTTGTTGCT
CmGEG-R1	TTAAGGGCACTTTCTCTTGCCACCATG
CmGEG-F2	CGCGGATCCATGGCCATCTCGAAGCCTTT
CmGEG-R2	GACGTCGACAGGGCACTTTCTCTTGCCA
pTRV2-CmGEG-F	CGCGGATCCATGGCCATCTCGAAGCCTTT
pTRV2-CmGEG-R	CCGGAATTCATGATAACTTACACCTAGCC
CmGEG-F3	CGGGGTACCATGGCCATCTCGAAGCCTTT
CmGEG-R3	CGCGGATCCTTAAGGGCACTTTCTCTTGCCA
qCmGEG-F8	GGCCATCTCGAAGCCTTTTGT
qCmGEG-R8	GTGCGCCTCTTTCCATCAAA
